# Supplementary material for: Study protocol for the Multimodal Approach to Preventing Suicide in Schools (MAPSS) project: A regionally based feasibility trial of an integrated response to suicide risk among UK secondary school pupils
Source: PLoS One. 2024 Jul 26;19(7):e0302873. doi: 10.1371/journal.pone.0302873 (PMC11280149; doi:10.1371/journal.pone.0302873)
Supplement: S1 Protocol — (DOCX) [file pone.0302873.s002.docx]

**S1 Protocol. Original Protocol Approved by Ethics Committee.**

**1. Full Title of Project**

Multimodal Approach to Preventing Suicide in Schools (MAPSS) project: A regionally based feasibility trial of an integrated response to suicide risk among secondary school pupils

**2. Background and Rationale**

**2.1 What is the problem being addressed?**

Rates of suicidal crisis among children and young people (CYP) are on the rise, with suicide rates per 100,000 adolescents having increased by 7-9% per year since 2010.^1^ Suicide is one of the leading causes of death in adolescents worldwide. In Northwest England, there has been an increase in attendances to Emergency Departments for CYP in suicidal crisis and/or for self-harm,^2^ and the number of CYP presenting is significantly worse than the UK average.^3^ Schools are a ‘universal access point’ for CYP, providing an opportunity to reach many CYP and intervene early. Suicide prevention interventions have been implemented in schools worldwide, but there are few in the UK and they have not been rigorously tested. The Multimodal Approach to Preventing Suicide in Schools (MAPSS) project, a suicide prevention intervention in Australia, has demonstrated feasibility and acceptability and is currently undergoing a randomised controlled trial (RCT) in Melbourne.^4^ The MAPPS intervention consists of three parts: suicide prevention lesson for all pupils, risk screening, and cognitive behavioural therapy (CBT) for those deemed to be at high risk for suicide ideation. However, cultural transferability of interventions cannot be assumed, and so we need to establish whether MAPSS could be effective in the UK.^5^ A recent scoping study^6^ of MAPSS conducted by the Co-PIs interviewed CYP, school staff, parents, and health professionals. All participants advocated the importance of school-based suicide prevention and gave feedback on the adaptations needed to the MAPSS intervention for the UK. The adapted MAPSS programme is being piloted across two schools to test the delivery, planning, and modifications needed. The proposed study aims to build on this work, employing a feasibility study design across six schools to assess: 1) the acceptability and safety of delivering MAPSS in a school setting in England; 2) the social validity (feasibility, utility, and acceptability) of the MAPSS intervention; and 3) the feasibility of delivering a large-scale, appropriately powered, cluster-RCT and economic evaluation of this intervention in the future.

**2.2 Why is this research important in terms of improving the health and/or wellbeing of the public and/or to patients and health and care services?**

This project aligns with the National Suicide Prevention Strategy’s critical actions and will contribute to the Zero Suicide initiative and the national awareness campaign by the ‘Three Dads Walking’.^7,8^ This year there has been increasing discussions about suicide prevention in schools following a petition by the Dads that received over 150,000, and the subsequent debate within Parliament (<https://commonslibrary.parliament.uk/research-briefings/cdp-2023-0060/>). The Baton of Hope are also developing an education charter highlighting how suicide prevention should be introduced into schools (<https://batonofhopeuk.org/>). Additionally, a national webinar hosted by The Jordan Legacy in England highlighted further the importance of including young people’s voices when designing and implementing suicide prevention curriculum content for schools (<https://thejordanlegacy.com/the-role-of-education-and-training-in-preventing-suicides/>).

Findings from the proposed study will help to inform the development and delivery of a suicide prevention intervention for CYP in schools, with the potential to improve lives and reduce the number of adolescent deaths. The cost of one suicide is approximately £1.5 million;^9^ thus, there are wider economic and societal benefits, including reduced costs associated with mental health difficulties across the life-course, and reduced strain on the NHS. In the Northwest, many CYP experience poor mental health, particularly children living in poverty. With public and patient involvement and engagement (PPIE) and stakeholder consultation, there will be enhanced interdisciplinary working, sharing of expertise and resources, and a greater sense of connectedness. Delivering the programme to 14-15 year olds provides an opportunity for early education and intervention. 50% of lifetime mental health conditions start by the age of 14 and 7.4% of 17- year-olds have previously attempted suicide,^10,11^ therefore intervening early may prevent early suicide attempts and improve adolescent mental health. This could incur long-term benefits into adulthood, as having a mental health condition and previously attempting suicide are both risk factors for future suicidal behaviours and service use.^1,12,13^

**2.3 How does the existing literature support this proposal?**

Suicide is the second leading cause of death in young people under 25 in England.^14^ Suicidal ideation and behaviour are associated with a host of negative outcomes including risk of future suicide.^15,16^ The impact of suicide on a young person’s family, friends, and wider community can be devastating, and also increases their own risk of suicide.^17^ There is therefore an urgent need to develop and test acceptable and effective approaches to preventing suicide in this population. Schools are an appropriate setting for the delivery of mental health prevention programmes, offering a ‘universal access point’ to all CYP, and have been identified as important locations for suicide prevention activities.^18^ Although school wellbeing staff may be able to provide support to pupils, CYP are often reluctant to seek help from professionals, preferring informal sources of support.^19^ School-based prevention efforts must therefore not only target school staff, but also fellow pupils.

Historically, there has been a reluctance to deliver suicide prevention efforts to pupils, due to concerns about potentially iatrogenic impacts.^20,21^ However, increasing evidence suggests that it is safe to do.^22,23^ A number of universal psychoeducation suicide prevention programmes have been tested internationally, and appear to increase knowledge, attitudes, and help-seeking intentions; some have also been associated with reductions in suicidal ideation and behaviour.^24,25^ Suicide prevention lessons such as safeTALK, a 3.5 hour lesson suitable for people age 14 and above (preventsuicide.org.uk), have been evaluated in youth populations.^18^ “Reframe IT”, an online Cognitive Behavioural Therapy (CBT) programme specifically designed for young people presenting in school with suicide risk, has also been found to be associated with reduced suicidal ideation, depression, and hopelessness in Australian CYP.^26,27^ According to international best practice, suicide prevention programmes should incorporate universal, selective, and indicated approaches.^28^ Such approaches have shown promise in both community and school settings.^29^ To date, only one study^4^ is applying rigorous economic methodology to evaluate short- and longer-term cost-effectiveness of an intervention comprising universal, selective, and indicated elements in schools.

A pilot study of MAPSS in two schools commissioned by Cheshire and Merseyside Public Health Collaborative (CHAMPS) has shown that it is feasible to recruit schools, have Papyrus deliver suicide prevention training to school staff and suicide awareness sessions to parents, for researchers to conduct surveys within the school setting at study timepoints, to identify pupils who may be at risk of suicide, deliver suicide prevention lessons with Year 10 pupils, and to recruit pupils to test the online CBT therapy programme, Reframe IT-UK. The pilot study will have been completed and written up prior to the start of this feasibility trial. Any modifications needed to the programme following on from the pilot will be discussed with the team and updated as required.

**3. Research Question**

Research question: What is the feasibility of a school-based suicide prevention programme comprising universal, selective, and indicated components in reducing suicide risk, improving risk recognition, and increasing health service use among young people aged 14-15 years in Northwest England?

MAPSS involves the delivery and evaluation of universal psychoeducation workshops (e.g., safeTALK) to school pupils, screening pupils for suicide risk (universal and selective approaches), and online CBT (Reframe IT-UK) delivered to pupils identified as being at-risk (indicated approach).

The primary aims of this study are to assess:

1. The acceptability and safety of conducting a trial of a suicide prevention programme in a school setting, operationalised in terms of adverse events and self-reported adverse consequences at post-intervention assessments.
2. The social validity (feasibility, utility, and acceptability) and implementation (including fidelity, quality, and dosage) of the MAPSS intervention, through a process evaluation consisting of bespoke quantitative surveys and qualitative interviews and focus groups.
3. The feasibility of delivering a large-scale, appropriately powered, cluster-RCT with economic evaluation in the future, including assessment of recruitment methods, retention rates, and outcome measures-and to assess links between these short-term trial outcomes and longer terms costs and outcomes.

We aim to test the following as secondary outcomes:

1. Comparison of suicide literacy and help-seeking intentions for suicidal thoughts or behaviours between the control group and pupils attending the suicide prevention workshop.
2. The identification of pupils at-risk of suicide who had not previously sought help.
3. Comparison of suicidal ideation, depression, and hopelessness between the control group and pupils in the Reframe IT-UK intervention group.
4. Rates of health-service use to inform an economic evaluation - levels of health-related quality of life.
5. Help-seeking intentions from informal sources.

**4. Research Plan/Methods**

**4.1 Design and Theoretical/Conceptual Framework**

The study will be conducted over a 2-year period (Jan 2024-Jan 2026), with 3 months for set up, 18 months feasibility, and 3 months consolidation.

This study adopts a multiphase feasibility design, consisting of 3 work packages (WP):

**WP1:** A three-month set-up stage to: i) make any adjustments to the interventions as determined from the pilot study, ii) recruit the RA, and iii) spend dedicated time recruiting schools from a diverse range of backgrounds, with two schools recruited in the first 3 months and the remaining four schools within 9 months, to enable alignment with school academic terms.

**WP2:** An 18-month feasibility study evaluating MAPSS in 6 schools: a feasibility cluster-RCT (schools as the unit of randomisation). Four schools will be randomised to receive MAPSS (intervention arm) and two schools will be randomised to continue with usual practice (control arm). Surveys will be completed by both arms at all timepoints. The trial will include i) baseline survey, ii) universal suicide prevention lesson (e.g., safeTALK) in intervention arm, iii) survey and screening 2 weeks after suicide prevention lesson, iv) an indicated Reframe IT-UK CBT intervention in intervention arm for pupils identified as high suicide risk, and usual care in control arm v) survey 2-weeks after Reframe IT-UK CBT, vi) survey 12-months post baseline.

**WP3.** A parallel process evaluation, to establish perceptions of social validity of the programme for use in the UK, and the appropriateness of the research design for effectiveness trials.

The study is facilitated by an extensive partnership with Local Authorities, schools, public advisors, professional educators, and researchers (see Letters of Support attached from collaborators and partner organisations). The specific objectives are to assess:

1. The acceptability and safety of a suicide prevention programme in schools

A mixed-methods approach will be used to determine acceptability and safety of trialling a suicide prevention programme in UK schools. The proportion of pupils who complete all agreed sessions of therapy will be recorded (>60% excellent; 40%-60% acceptable; <40% not acceptable).

Safety will be operationalised in terms of adverse events linked to the intervention (e.g., suicidal behaviour, crises, or increased self-harm), and in terms of self-reported adverse consequences at post-intervention assessments.

1. The social validity of MAPSS

Participants will complete bespoke social validity measures adapted from the Australian trial.^4^ We will also undertake a qualitative process evaluation, consisting of semi-structured interviews and focus groups in four ‘case study’ schools (see further details in section 4.7) with pupils, in addition to as many pupils as possible who dropout (estimated to be a much smaller group), to determine their experience of MAPSS, what was helpful or not helpful, and problems in completing sessions. We will also carry out interviews with teachers and members of the senior leadership team to investigate facilitators and barriers to delivering MAPSS within school. Module utilisation, attendance at suicide prevention lessons, and screening will be monitored to review whether all aspects of the programme are used and when and where they are used.

1. The feasibility of delivering a large-scale trial of this intervention in the future

Uptake and retention of participants in school-based intervention trials is a major issue that impacts the feasibility of future trials.^30^ This will be monitored through a mixed-methods evaluation encompassing quantitative and qualitative indicators. Quantitative indicators will include: a) comparisons of recruitment methods (for schools and then pupils into the study); b) successful data collection rates (low missing data); c) presence of floor/ceiling effects in collected data; d) whether responses on potential outcome measures remain static (or show ‘movement’) between baseline and follow-up; e) calculation of the precision of estimates of key parameters, which will inform a future trial. Qualitative indicators will include evaluation of the perceived mechanism of change and contextual factors impacting upon the effectiveness of the intervention for pupils and teachers. We will seek to interview participants who do not complete follow-up questionnaires to determine their reasons for not doing so and any factors that would improve completion rates. Factors that will determine stop/go procedures to a definitive trial will include: evidence that recruitment to a definitive study, using optimum recruitment pathways in one region, would be as follows: number of schools recruited at 9 months (6 excellent, 3-4 acceptable, 1-2 unacceptable), number of high-risk pupils recruited in the cluster-RCT per school (10-15 excellent, 5-10 acceptable, 0-5 unacceptable), and proportion of complete data collected (>80% acceptable). Retention will be judged by the following criteria: >80% completion excellent; 60-80% acceptable; below 60% unacceptable.

**4.2 Progression Criteria**

A set of eight provisional progression criteria for the MAPSS programme have been established to determine whether a full RCT is warranted, and will be further developed in collaboration with the independent Trial Steering Committee (TSC; see Table 1). All progression criteria will need to be met for the MAPSS programme to be seen as acceptable and feasible, and to progress to a full efficacy trial.

*Table 1. Traffic light feasibility progression criteria*

| **Progression Criteria** | **Red**  **(stop)** | **Amber**  **(discuss and amend)** | **Green**  **(go)** |
| --- | --- | --- | --- |
| School recruitment (targeting n=6) | 1-2 schools recruited | 3-4 schools recruited | 6 schools recruited |
| Pupil participant recruitment (targeting n~810) | <20% of eligible pupils | 20-74% of eligible pupils | ≥75% of eligible pupils |
| School staff training recruitment | ≤2 teachers per school | 3-6 teachers per school | ≥6 teachers per school |
| Suicide Prevention workshop | <80% of scheduled workshops delivered | 80-99% of scheduled workshops delivered | 100% of scheduled workshops delivered |
| Pupils screening at high-risk of suicide | <5 pupils per school | 5-10 pupils per school | 10-15 pupils per school |
| Reframe IT-UK  Online CBT | <40% of eligible pupils engage with ≥75% of modules | 40-69% of eligible pupils engage with ≥75% of modules | ≥70% of eligible pupils engage with ≥75% of modules |
| Acceptability of intervention | <50% of pupils found MAPSS acceptable | 50-79% of pupils found MAPSS acceptable | ≥80% of pupils found MAPSS acceptable |
| Outcome data collected at baseline | Data collected from <50% of pupils | Data collected from 50-79% of pupils | Data collected from ≥80% of pupils |
| Follow-up outcome data attrition at T3 | >40% data attrition at T3 | 21-40% data attrition at T13 | ≤20% data attrition at T3 |

**4.3 Planned intervention**

The training in schools for pupils, teachers, and parents will be funded by suicide prevention commissioners within the Local Authority (see Director of Public Health Letter).

1) A minimum of 6 staff from each school will receive training from Papyrus: **Suicide Prevention – Overview Tutorial (SP-OT)**, to ensure staff are equipped to manage any risk identified from the screening. SP-OT is delivered in a single session over 1.5 hours online. Papyrus will also provide an online information session, **Suicide Prevention - Awareness, Resource, Knowledge (SP-ARK),** along with support packs, for parents of all children in Year 10 at each participating school. #MyGPGuide; a guide for CYP with lived experience of self-harm and suicidality will be shared with schools and families.^31^

2) **Suicide prevention lesson** such as safeTALK, developed by LivingWorks Education,^32^ a suicide alertness training workshop suitable for anyone over the age of 14. The lesson comprises a single 3.5-hour face-to-face workshop, designed to help participants understand suicide warning signs in themselves and others, gain knowledge about sources of support, apply basic ‘TALK’ steps (Tell, Ask, Listen, and KeepSafe), and signpost others. Suicide prevention lessons will be delivered by trained Suicide Prevention Facilitators at Grassroots Suicide Prevention to classroom-sized groups of pupils (maximum 30 pupils per session with at least one teacher present).

3) The **screening** will take the form of self-report measures embedded into the questionnaires at each timepoint. Researchers will inform each school after each timepoint of any pupils who are assessed to be at risk. Pupils who report suicidal ideation within the past four weeks (Suicide Ideation Attributes Scale [SIDAS] score of 21 or higher)^33^ or any level of current suicidal ideation (single multiple-choice item) will be flagged by the research team and followed up by the school safeguarding lead.

4) **Reframe IT-UK** has been adapted from the Reframe-IT intervention developed in Australia.^17,27^ It comprises eight 20-minute online self-guided CBT modules, following the stories of two young people who make video diaries about their day-to-day life and their experience of feeling suicidal. There is also a message board through which the participants can communicate with a moderator, a mood diary, and signposting information.

**4.4 Control/Comparator Group**

Participants in schools randomised to the control group will receive treatment as usual (TAU; e.g., from the school nurse or external mental health services), based on the typical provision at each school. The pastoral staff will be asked to record what TAU comprises in each school through the completion of a ‘usual practice’ survey prior to randomisation and at T4, to establish programme differentiation, any changes over time, and to control for any compensatory rivalry that may occur over the course of the trial. To ensure safety and appropriate support in the event of pupils being flagged as at-risk in the control group, schools who do not engage with the SP-OT (suicide prevention training for designated teachers in recruited schools) training will be unable to progress through the trial.

**4.5 Setting/Context**

Mainstream secondary schools and Pupil Referral Units (PRU) in Northwest England will be approached to participate. Papyrus’ SP-OT training for teachers will be delivered in all schools. The suicide prevention lesson will be delivered in all 6 schools (4 in the intervention arms and 2 in the control arm after the trial is complete, *if deemed safe*), in typical classroom settings. Pupils participating in Reframe IT-UK in the 4 intervention schools will complete the modules online during school time, in the presence of a pastoral member of staff, with the option of accessing additional resources during their own time. A guidance pack for schools will is being developed in collaboration with Headteacher Co-Is (SY) and a manual will be provided for Reframe IT-UK. The guidance will be provided to participating schools and will advise them of key information, including project timelines and milestones, their role and responsibilities, and young people’s rights throughout the project (including the importance of safeguarding and confidentiality). It will also include information on supporting staff wellbeing.

**4.6 Study Population**

Participants will include Year 10 pupils (aged 14-15). Opt-out ethical procedures will be used – these have been approved by the university research ethics committee in the pilot study and were acceptable to parents and schools. We will utilise purposeful maximum variation sampling to recruit schools in different areas across the region and to ensure inclusivity of diverse populations (e.g., rural/urban, ethnic minority groups, differing deprivation levels, differing academic achievement levels). We will work with partners across the region who can support with this (e.g., Papyrus, Cheshire and Merseyside Safeguarding Group).

The sample will consist of ~810 adolescent pupils in Year 10, recruited from 6 mainstream secondary schools across Cheshire and Merseyside. Six schools will be randomly assigned to one of two arms as part of a cluster-RCT: intervention (n=4) or control arm (n=2). Year 10 pupils in the intervention schools will receive: 1) suicide prevention lesson (n~540 pupils); and 2) pupils scoring 21 or above on the SIDAS^33^ or indicating past suicide ideation will also be offered Reframe IT-UK plus TAU (n~54 pupils). Those in the control arm will receive TAU only (n~27 pupils).

**4.7 Overview of Research Methods**

***Quantitative Data Collection***

Participants will complete a suite of quantitative measures (see details below) online in school at 4 time-points: baseline (T1); 2-4 weeks post-baseline (after suicide prevention lesson; T2); 12 weeks post-baseline (after Reframe IT-UK; T3); and 1-year post-baseline (T4). The survey system will flag participants who score in the at-risk range for suicidal ideation.^33^ The research team will then contact the school about these pupils to determine eligibility for participation in the Reframe IT-UK in intervention schools. Eligible pupils will then be offered the intervention and TAU. Pupils receiving the Reframe IT-UK intervention will complete the 8 modules in the 10 weeks between T2 and T3 (i.e., approximately one module per week). Primary outcomes will be measured at T2 for suicide prevention lesson and T4 for Reframe IT-UK.

***Qualitative Process Evaluation***

We will conduct longitudinal case studies of the 4 schools randomised to receive MAPSS. The case studies will explore inter-related issues of 1) social validity of MAPSS and 2) *how* MAPSS was implemented and *why* it was implemented in this way. In terms of social validity, we will utilise Wolf’s framework,^34^ focusing on key tenets of acceptability, feasibility, and utility (e.g., does the intervention meet schools’ perceived needs? How well received is the intervention among staff and pupils? Can the intervention be delivered successfully?). Here we will draw upon relevant studies of school-based interventions (e.g., Kendal, et al)^35^ and adapt existing rubrics from the implementation literature (e.g., Bird et al)^36^ to inform our data generation.

In terms of how MAPSS was implemented, we will focus on the following dimensions: fidelity (e.g., to what extent teachers adhered to MAPSS guidance), dosage (e.g., how much of MAPSS pupils accessed), quality (e.g., how well MAPSS was delivered), participant responsiveness (e.g., the extent to which pupils engaged), reach (e.g., the rate and scope of participation), programme differentiation (e.g., to what extent MAPSS can be distinguished from other, existing mental health programmes), and adaptations (e.g., the nature and extent of changes made during implementation). We will also explore a range of factors that may have affected implementation at the different domains/levels consistently: preplanning and foundations (e.g. buy-in), implementation support system (e.g. ongoing external support), implementation environment (e.g. time constraints), implementer factors (e.g. experiences, skills and confidence in delivery), and programme characteristics (e.g. flexibility).^36-38^

***Methods for Sharing Study Progress and Findings with Participants and Payments, Rewards and Recognition for Participants***

Each participating school will be invited to nominate a minimum of 6 key staff members to complete SP-OT training delivered by Papyrus, to increase staff capacity to enact safety protocols, and to act as an incentive for schools to participate. To ensure safety and appropriate support in the event of pupils being flagged as at-risk, schools who do not engage with the SP-OT training will be unable to progress through the trial.

Upon completion of the study, schools will be provided with an anonymised aggregate report of the questionnaire data collected to encourage retention and to recognise the time they have given to the study. Schools will also be provided with a certificate of participation.

**4.8 Sampling**

To ensure a diverse range of schools and CYP are included in the feasibility trial, we propose purposeful maximum variation sampling. This is widely used in research to pragmatically identify and select participants that are effective in addressing the research aims, while also maximising diversity and limiting bias.^39^ The key characteristics we would seek variation on include: rural/urban status, proportion of ethnic minority pupils, schools’ deprivation levels (IDACI), schools’ academic achievement (proportion of pupils achieving benchmarks GCSE grades). To ensure we achieve this, we have allocated dedicated time in the project plan during the setup phase to collate school data (publicly available), approach and build relationships with schools who do not typically engage in research, and ensure buy-in from the senior leadership teams (via the collaborators). Both Principal Investigators have expertise in involving diverse communities in research programmes and have started this process during the pilot study.

*Sample size calculation:* As this is a feasibility study, a sample size calculation is not needed. In line with similar work,^26-28^ we will recruit 6 schools to provide sufficient variety of schools and pupil numbers in which to test recruitment, retention and acceptability of the intervention, and feasibility of the research design for evaluation. This is based on the assumption of a potential attrition rate of 50% at T3 (the primary outcome point for Reframe IT-UK). This is common for feasibility trials,^40^ realistic in terms of recruitment, and would allow adequate precision in estimating rates (e.g., attrition, adverse events) relevant to trial outcomes.^40^ This would allow an overall attrition rate of 50% to be estimated with 95% confidence intervals of +/- 12% or, 16% for a single arm. This sample size is also adequate for estimating relevant analysis parameters such as the standard deviation of effects, which are needed for determining the feasibility of a later efficacy trial.^40,41^ Based on findings from the pilot study, we anticipate that 10% of pupils will score in the at-risk range in the screening and will thus be eligible for Reframe IT-UK.

We will monitor recruitment rates from the different methods and associated costs by collecting data on:

1. The proportion of eligible young people who consented;
2. The number of participants recruited during the recruitment stage of feasibility compared with the target;
3. Assessment of contamination of MAPSS programme in control schools;
4. Assessment of CYP satisfaction with intervention and outcome measures.

This will provide evidence on recruiting to trials in school settings, as well as informing the full trial design.

**4.9 Randomisation**

The unit of randomisation is the schools. After baseline data collection is complete, 6 schools will be randomised to one of two study groups. We will be comparing both arms to test which, if any, is better. To create balance in terms of deprivation, ethnicity, rurality and educational outcomes, a minimisation algorithm will be used at an intervention-to-control ratio of 2:1 across Cheshire and Merseyside. Two schools will be allocated to the control arm and four schools will be allocated to the intervention arm by a university statistician, who is independent of the study and blind to school identities. Methods of allocation concealment and randomisation processes will follow CONSORT^42^ (see figure 1). Schools will be randomised to receive a suicide prevention lesson (e.g., safeTALK) and Reframe IT-UK and TAU, or TAU only, via a random sequence generation computer algorithm. The method of randomisation will be conducted using a routine within STATA to generate (stratified) randomised (block sizes of 2, 4 and 6) allocations. Researchers completing study assessments will be masked to intervention allocation. The trial will follow an Intent-To-Treat (ITT) protocol. Attrition will be recorded and reasons for drop-out recorded where possible. Guidance will be sought from the advisory groups on how to manage and minimise attrition over the study period.

**4.10 Outcome Measures** **(Quantitative)**

***Primary Outcome Measures***

***Acceptability:*** Operationalised in terms of acceptability of the intervention. The proportion of pupils who complete all agreed sessions will be recorded (>60% excellent. 40%-60% acceptable; <40% not acceptable) on the Reframe IT-UK website. Acceptability of the suicide prevention lesson, including whether or not participants thought it was “useful”, “interesting”, or “upsetting”, will be assessed at T2 only using purpose designed items. Participant views on the Reframe IT-UK intervention will be assessed at T3.

***Social validity:*** We will test this through a process evaluation using bespoke quantitative surveys following the delivery of each component of the MAPSS programme at T2 and T3. We will also conduct qualitative interviews with staff and focus groups with pupils across the study period.

***Feasibility of the trial:*** We will collect data on: 1) the missing data on completed assessment (<15%); 2) change or variability on outcome measures (e.g., suicide ideation, depressive or hopelessness symptoms); and, 3) whether schools implemented and supported the accessibility of the online intervention.

***Secondary Outcome Measures***

The secondary outcomes for this study are:

1. Change in **past four-week suicidal ideation** at T3 and T4, compared to T2, assessed via the SIDAS.^33^ The SIDAS is a self-report measure designed to screen individuals in the community for presence of suicidal thoughts and assess the severity of these thoughts.
2. Change in **symptoms of depression** at T3 and T4, compared to T2 and T1, will be assessed using the Patient Health Questionnaire – 9 item version.^43^
3. Changes in **hopelessness** at T3 and T4, compared to T2, will be assessed using the Brief-H-Pos, a 2-item positively worded measure of hopelessness.^44^
4. Differences in **health service use** and other resource use (education and local authority) comparing intervention and control group at T2, T3 and T4, will be assessed using a bespoke questionnaire adapted from the Young Mind Matters Service Use questionnaire.^45^
5. Purposefully designed questions on **intentions to seek help from informal sources**.
6. Change in **health-related quality of life** during the trial (T1, T2, T3, T4) will be assessed using the Child Health Utility–9 (CHU9D).^46^ The CHU9D can be used to derive quality-adjusted life years (QALYs). These data will be used in combination with data from 3) above, to assess the feasibility of the full economic evaluation.
7. Change in **suicide literacy** at T2, T3 and T4, compared to T1, will be assessed using an adapted version of the Literacy of Suicide Scale (LOSS).^47^

School staff (key contact or safeguarding lead) will complete a usual practice survey at T1 and T4, to ascertain current provision (i.e., establish a clear counterfactual), identify the level of programme differentiation, and to account for any potential compensatory rivalry or contamination in control schools.

**4.11 Methods for Data Collection**

***Quantitative Data Collection***

Surveys will be completed online during school time by pupils. Schools will be asked to book an IT room for pupils to complete the surveys and will be provided with a detailed support pack for completing the measures with pupils, including links to the surveys, age-appropriate lesson plans, PowerPoint slides, and glossary. Surveys will not be anonymous as pupils will need to be monitored for risk and screened for potential participation in Reframe IT-UK. Thus, data linkage across time points will not be an issue. A ‘usual practice’ survey will be completed at T1 by one staff member at each school, to determine what ‘usual care’ looks like, and levels of program differentiation.

***Qualitative Data Collection***

As part of the qualitative process evaluation, case study fieldwork visits will be conducted at T2 (after suicide prevention lesson) and T3 (after Reframe IT-UK). We will use semi-structured interviews with school staff and intervention deliverers (N=3 per school x 2 visits = 8 interviews), and interviews (for Reframe IT-UK) and focus groups (for suicide prevention lesson) with pupils (N=1 per school x 1 visit = 4 focus groups; N=2 per school x 1 visit = 8 interviews), as well as observations and document analysis of intervention delivery.

Class teachers and members of the senior leadership (e.g., safeguarding leads) will be interviewed individually at each case study visit. Small groups (n=4-6) of pupils will participate in semi-structured focus groups regarding suicide prevention lesson (to reduce power imbalances and ease nerves), and one-to-one interviews (with a teacher present if requested) will be conducted with pupils who have taken part in Reframe IT-UK (due to the sensitive and personal nature of intervention participation). Bespoke semi-structured interview schedules have been developed for each key stakeholder group. All interviews/focus groups will cover trial feasibility and acceptability, and factors affecting implementation; overarching this will be a social validity framework.^48^ However, each schedule will be tailored to the relevant time point and stakeholder group. Prompts and probes will be utilised where necessary to clarify unclear responses and elicit further detail. Interviews and focus groups will be conducted in private and quiet parts of the school, and fully informed consent will be ensured.

Professionals who delivered the interventions in the case study schools will also be invited to be interviewed, to ascertain fidelity, quality, and dosage, and gain their perspectives on participant engagement and reach, as well as the feasibility of an efficacy trial. Interviews will be conducted at a time and place to suit them (face-to-face or online). Observations and document analysis will be arranged where possible with the intervention deliverers for additional context. All interviews/focus groups will be audio recorded and transcribed verbatim.

**4.12 Data and Statistical Analysis**

***Quantitative Data Analysis***

Changes in primary and secondary outcomes over time will be assessed for pupils clustered within schools and the treatment group included as a fixed factor. The analysis will adopt the intention-to-treat approach. As a secondary analysis, compliance (as measured by the number of modules completed) and other potential covariates (e.g., gender) will be accounted for. If the amount of missing data is non-trivial, multiple imputation will be used. We will follow the recently published extension to CONSORT^42^ when reporting the results of this feasibility trial. We will calculate and present in a CONSORT flow chart:

- - 1. The proportion of young people with suicide ideation consenting to the study;
    2. The proportion completing baseline assessment and entering the randomised phase;
    3. The number of online modules used and the proportion completing all sessions;
    4. The proportion completing follow-up assessments at 12-weeks and one-year post-randomisation.

As this is a feasibility study, no formal hypothesis testing will take place. Quantitative data will be reported using summary statistics and 95% confidence intervals. We will compare the continuous scores on the SIDAS outcome measure graphically to assess between group trends. The SD of the primary outcome will inform the sample size calculation for the large-scale RCT.

***Qualitative Data Analysis***

Qualitative data will be treated in two ways. First, we will produce detailed case profiles of each school that document their implementation, paying attention to how individual context and circumstances have influenced progress in each. Secondly, interview and focus group transcripts will be analysed via thematic analysis using the framework approach.^49^ A hybrid approach will be taken, which will be informed by conceptual models of implementation in school settings^50^ and our primary orienting concepts (social validity, acceptability, feasibility), while allowing for unanticipated themes specific to this project/context. We will also adopt Normalisation Process Theory (NPT)^51^ as a broad framework through which to make sense of the qualitative data and draw conclusions relating to how readily MAPSS might be implemented amongst schools and embedded into school systems.

The qualitative framework analysis approach was developed to meet information needs and to provide outcomes or recommendations.^52^ It offers a highly visible and systematic approach to data analysis, showing very clearly how findings are derived from the data. This approach also facilitates analysis of specific concepts and issues that are particularly important to address, and so facilitates the use of NPT in interpreting the data. Analysis will follow the five suggested stages of framework analysis (Familiarisation with the data; Identifying a thematic framework; Indexing the data; Charting the data; Mapping and interpretation).^53^ In order to monitor and limit the impact of a single perspective, PS will examine parts of the transcripts to compare their perceptions of the interview data and analysis with the analyst’s interpretation. Further data analysis will be completed in research team meetings with the RA, PM, EA, TN, DC, LH and PS.

NPT provides a framework for understanding the barriers and facilitating processes that underlie the implementation and integration of complex interventions into systems.^53,54^ The theory has been developed from qualitative research and identifies four key processes that underlie the adoption of new interventions (coherence of intervention; cognitive participation; collective action; reflexive monitoring). Previous research has shown that NPT can be applied effectively to qualitative data in healthcare contexts and, more recently, in school-based research.^55^ NPT will be drawn upon as a putative framework within the qualitative analysis, and an attempt will be made to map the links between qualitative themes arising from the data and the core processes outlined in NPT. This process will be aided through use of the NPT toolkit (<http://www.normalizationprocess.org/>) and application of May et al.’s^53^ NPT statements. in order to further promote integrity and rigor during the analysis process, field notes will be written immediately after interviews and a reflective diary maintained.^56^

**4.13 Health Economic Evaluation**

We will collect data to assess the feasibility of conducting a full cost-effectiveness analysis in a future study. Initially we will consider an NHS and Personal Social Services perspective but will explore the possibility of expanding this to incorporate impacts on other (non-health) sectors using the resource use questions described in section 4.10 (for example education and Local Authority resource use). For this feasibility study, participants will complete the CHU9D health service resource use questionnaire,^46^ and other instruments described above. Data will be collected on resource use required to deliver the service (e.g., the time required for staff to deliver the intervention) to estimate the cost of the intervention. Data required to estimate health-related utility and quality-adjusted life-years (QALYs) in a subsequent trial will be collected using the CHU9D. QALYs will not be calculated in this feasibility study; rather, disaggregated data based on health-related resource use data and health related quality of life (CHU9D) for intervention and control groups will be presented.

We will conduct a review of economic models to assess whether there are other comparator interventions and to establish a link between the short-term outcomes of this trial (suicidal ideation/mental health) to longer term costs and health-related quality of life. This will inform the longer-term model conceptualisation that will form part of the economic evaluation alongside the main definitive trial, but will also provide a useful assessment of the longer-term impact of these short term outcomes. In the main trial, we will assess the impact of inequality on cost-effectiveness (that is, establishing on which groups the main costs and effects impact) using Distributional Cost Effectiveness Analysis (DCEA).^57^ In this feasibility study, we will assess which variables (e.g., socio-economic status) can be used as categories in the DCEA.

**4.14 Assessment and Follow Up**

***Assessment of unanticipated outcomes:*** A MAPSS Safety Monitoring Committee (SMC) as part of the TSC will be established (members of the steering group and research team) as the main vehicle for both safety and investigator oversight of the MAPSS project, including data monitoring, endpoint adjudication, and data management strategy. This committee’s composition and charter will be described in the co-produced MAPSS Safety Monitoring Plan. It is comprised of the Co-Is and other representatives of the named investigators, as well as independent internal and external subject matter experts including young people. The composition of the group is designed to incorporate transparency and ensure that no one set of competing interests can unduly influence other stakeholders and is appropriate for this non-commercially funded study. This committee has a dual safety role: it incorporates a risk-appropriate safety, endpoint adjudication and data management strategy which is responsive to study issues as they eventuate. A formal Data Safety and Management Committee (DSMC) will be convened if and when the SMC deem this escalation is required. A comprehensive safety protocol is being co-developed, which will be activated if: 1) participants return a score of 21 or higher on the SIDAS at any time-point; 2) participants report current suicidal ideation at any time-point; 3) participants report suicide risk via the Reframe IT-UK platform. Ultimately all risk information will be communicated to the school, who will be responsible for ongoing management. Adverse events (AEs) or serious adverse events (SAEs) that arise during the trial will be recorded in the study database.

An AE is the development of an untoward effect, undesirable clinical occurrence or medical condition, or the deterioration of a pre-existing medical condition following or during exposure to a study intervention, whether or not considered causally related to the study intervention. For the purposes of safety reporting, any research activity is considered to be part of the “study intervention”. An AE can therefore be any unfavourable and unintended clinical sign, symptom, observation, or disease temporally associated with the use of an intervention, whether or not related to the intervention. An SAE is any untoward medical occurrence that: results in death or is life-threatening (‘life-threatening’ in the definition of SAE refers to an event in which the participant was at risk of death at the time of the event, it does not refer to an event which hypothetically might have caused death if it were more severe); requires inpatient hospitalisation or prolongation of existing hospitalisation; results in persistent or significant disability/incapacity; is an important medical event that although not immediately life-threatening or result in death or hospitalisation, based upon appropriate medical and scientific judgment, may jeopardise the participant and/or require intervention to prevent one of the outcomes listed above. Outpatient treatment in an emergency department is not in itself an SAE, although the reasons for it may be (e.g., suicide attempt). Hospital admissions and/or surgical procedures planned before or during a study are not considered SAEs if the illness or disease existed (or the surgery was planned) before the participant was enrolled in the study, provided that it did not deteriorate in an unexpected way during the study.

***Assessment and documentation of AEs:*** All AEs and SAEs that arise during the trial will be recorded in the study database. The causality of AEs and SAEs (i.e., their relationship to intervention treatment) will be assessed by a suitably qualified study team member. Any SAE will be reported to the Sponsor and to the relevant ethics committees within 24 hours of the research team becoming aware of its occurrence.

**4.15 Scalability and Translation**

Scaling up effective health interventions is vital as not doing so could deny communities of the most effective services and programmes.^58^ We will utilise the Intervention Scalability Assessment Tool (ISAT) that was developed to support researchers, policymakers, and practitioners to make systematic assessments of the suitability of health interventions for scale-up.^58^  This will include three parts:

- ***Part A*** ‘setting the scene’ requires consideration of the context in which the intervention is being considered for scale-up and consists of five domains, as follows: (1) the problem; (2) the intervention; (3) strategic/political context; (4) evidence of effectiveness; and (5) intervention costs and benefits.

- ***Part B*** asks users to assess the potential implementation and scale-up requirements within five domains, namely (1) fidelity and adaptation; (2) reach and acceptability; (3) delivery setting and workforce; (4) implementation infrastructure; and (5) sustainability.

- ***Part C*** generates a graphical representation of the strengths and weaknesses of the readiness of the proposed intervention for scale-up. Recommendation as to whether the intervention (1) is recommended for scale-up, (2) is promising but needs further information before scaling up, or (3) does not yet merit scale-up.

The scalability and translation of the MAPSS intervention into wider contexts if proven to be effective will depend on the feasibility of implementing the components within the trial and the acceptability from schools and pupils. The logic models (see attached) provide the theory of how the two main components of the intervention produces its outcomes; thus providing the ‘theory of change’ about how the suicide prevention lesson and Reframe IT-UK interventions work. The logic model will help to track and monitor operations within the trial to better manage results. This will help to create a foundation for creating budgets, work plans, and improving communication as part of the scalability and translation for scaling-up.

**4.16 Socioeconomic Position and Inequalities**

As part of the scoping study conducted to inform the adaptation of the intervention for the UK, we used the health inequalities assessment tool (HIAT – FOR-EQUITY; forequity.uk)^59^ to ensure that we addressed inequalities. The HIAT aims to support researchers (and stakeholders) to integrate an intersectional equity lens into research and consider how people with lived experience and policy or practice expertise can help in this process. We have also since completed the NIHR Equality Impact Assessment. From the outset, we ensured that we involved parents from different cultural backgrounds and CYP from schools in communities with different levels of deprivation. We do not anticipate pupils being excluded from taking part in the MAPSS programme as it will be commissioned by Local Authorities as a school-based workshop. However, parents/carers will have received notification about the workshop and will be able to opt their children out of attending. We will record demographic data for pupils who are not consented to attend the suicide prevention lesson or those not consented to take up the Reframe IT-UK intervention, to review the reasons of why this may be. We will be adopting purposeful maximum variation sampling to recruit schools and have dedicated time in the study to ensure we recruit a diverse sample (see section 4.6 above further details). We have also adapted the materials as part of the Reframe IT-UK intervention, which includes video diaries recorded by young people (actors). We have ensured that actors and their stories are diverse and reflect the socio-demographic characteristics of UK schools, and we have worked with young people to develop these. So far, we have not needed to translate study materials for Year 10 pupils in mainstream secondary schools in the UK. However, if we do encounter such issues, we will record and report these findings to inform the design of future trials. Findings from our scoping study suggested that some elements of MAPSS were not accessible for neurodivergent pupils, and recommended adaptations were implemented for the pilot study (e.g., Reframe IT-UK is now completed with a pastoral member of staff, the workshop has more breaks). However, we have been monitoring this throughout the pilot study, and will make any further adaptations (if necessary) for the feasibility trial.

**5. Dissemination, Outputs, and Anticipated Impact**

**5.1 What do you intend to produce from your research?**

Project findings will be shared through reports developed in close consultation with schools, NHS professionals, public health and third sector organisations, and those affected by suicide. Findings will be of interest to various stakeholder groups, and so bespoke reports will be developed for education, health and social care organisations, and researchers in the field. Outputs will include:

- Publications in high impact peer-reviewed journals
- Presentations/symposia at national and/or international conferences
- Summary briefs for different audiences
- Policy evidence briefings
- Public-facing website, including short videos/animations, infographics, and blogs/vlogs to highlight the work.

**5.2 How will you inform and engage patients/service users, carers, NHS, social care organisations and the wider population about your work?**

We are aiming to have a real-world impact by collaborating with clinicians, PPI members, academics, and third sector organisations. The outcomes from the trial are important to both clinical practice and research as they help practitioners understand what they are doing, how effective it is and improve understanding about this specific patient group. Our impact strategy will be carefully designed to maximise impact and dissemination of results across the main stakeholder groups affected by this research. The main impact goals are to:

1. Contribute to the body of knowledge on effective school-based suicide prevention intervention in schools.
2. Influence public bodies and policymakers on implementing school-based suicide prevention interventions in schools, whereby an intervention can take place within a child’s own community, including more deprived communities where suicide ideation appears more prevalent.
3. Contribute to economic development by reducing admissions to Emergency Departments for suicidal behaviours by providing quicker psychological interventions within school settings.

For dissemination to be effective, dialogue is needed with relevant audiences. Project findings will be disseminated in close consultation not only with academics but also with schools, clinicians, community mental health professionals, public health, third sector organisations (e.g., PAPYRUS), and those affected by suicide behaviours in young people.

The work will be of considerable interest to education, social, clinical, and academic professionals in the field of suicide prevention and community mental health. Publications in renowned, high-impact journals, alongside presentations at regional, national, and international conferences will be pursued to maximise dissemination amongst academic and research audiences. A high specification executive summary of the key findings will be disseminated to schools, clinical practitioners, and researchers across the UK.

A one-day national conference will be hosted, funded jointly (by organisations working on this project), focusing on dissemination and discussion of project findings. Academics, researchers, schools, third sector organisations, social and clinical service staff, and the public will be invited, ensuring a range of perspectives are present on the day. The one-day conference will be used as a platform to gain initial interest from NHS England Public Health Suicide Prevention Leads across England, through which future engagement can then be supported. Within the conference we will ensure that individuals and carers affected by suicidal behaviours are participating within the programme; their voices will be actively encouraged and listened to in considering the development and implementation of the subsequent trial.

Press releases at key project milestones will be disseminated via an ongoing social media campaign, designed to further disseminate project progress and findings. Summary and guidance documents will be created and made available to schools managing pupils with suicidal behaviours via the study website page. The next step of the research (efficacy RCT) will be supported by a pro-active engagement with schools across the region via NHS England Public Health Suicide Prevention Leads and the CRN.

**5.3 How will your outputs enter our health and care system or society as a whole?**

The role of the partner organisations will be vital in enabling MAPSS to be adopted by the Department of Education (DfE), who oversee the delivery of school-based programs across England, and NHS England who would commission school-based suicide prevention programmes. Both the DfE and NHS England recognise the lack of suicide prevention programmes directly targeting school pupils, as well as gaps in the evidence regarding the efficacy of suicide prevention interventions. If effective, Reframe IT-UK will be made available to school staff to use with at-risk pupils through LJMU’s Suicide and Self-Harm Research Group, and suicide prevention lessons are embedded into Grassroots’ core educational programs with the capacity to be rolled out nationally via existing networks. A member of the DfE (CN) sits on the TSC and will feed this information into educational policy meetings.

**5.4 What further funding or support will be required if this research is successful (e.g. From NIHR, other Government departments, charity or industry)?**

The training in schools for students, teachers, and parents will be funded by Local Authority suicide prevention commissioners, where suicide prevention in schools is now one of their top three priorities (Total = £27,630). This includes online training by Papyrus for staff in schools (SP-OT; £600) and parents (SP-ARK; £480), along with administration (£240) and resource packs for parents (£360) = £1680. Suicide prevention lessons will be delivered by Grassroots at £25,950 across 6 schools. Leads of the suicide prevention groups are included as a co-investigator (NB) and collaborators (RdP).

**5.5 What are the possible barriers for further research, development, adoption and implementation?**

Possible barriers for future research, development, adoption, and implementation include:

1. The reliance on funding from local commissioners to fund the intervention in schools across the region/nationally.
2. Trained specialists such as Grassroots being available to deliver the suicide prevention lesson across schools region/nationally.
3. Trained specialists such as Papyrus being available to train teachers in suicide prevention in schools and to provide online information sessions to parents.
4. Obtaining the required License agreement for use of Reframe IT in UK schools as the adapted Reframe IT-UK.

**5.6 What do you think the impact of your research will be and for whom?**

This project responds to the rising rates of suicide among young people in the UK. These rates are concerning, particularly for school staff who are often the first point of contact for at-risk pupils yet do not always feel confident to respond. This project is the first feasibility study in the UK to test an integrated approach to suicide prevention in secondary schools and, in doing so, aligns with international best practice and national and regional policy priorities.^6,7^ It is also unique in that it involves educating young people directly about suicide and will test an innovative online programme with pupils identified as at-risk. The project represents a strong and well-established partnership between the research team, the DfE, NHS England Public Health Leads, Grassroots Suicide Prevention Charity, Papyrus, the Martin Gallier Project, and participating schools; a partnership that has capacity-building and the transfer of knowledge into practice and policy at its core.

The Reframe IT-UK programme will deliver evidence-based treatment to pupils identified as at-risk, potentially reducing distress and preventing future suicidal behaviours. If identified to be effective in future trials, it will be made readily available to schools, further improving the lives of thousands of CYP and helping to reduce the number of young deaths by suicide. Furthermore, the evaluation of the suicide prevention lesson will lead to the education of almost 810 English secondary school pupils under the auspices of this project alone, improving suicide literacy, help-seeking knowledge, and coping skills. If effective, suicide prevention lessons will become embedded into Grassroots’ core programmes, meaning that, if supported by the DfE, they could be rolled out nationally to schools across the country. As a result, this partnership has the capacity to provide evidence-based suicide prevention education to tens of thousands of young people in an ongoing way. This, together with the fact that training will also be provided to nominated school staff and parents, means that the project will increase capacity across all participating schools and potentially the wider community. The individual researchers and the partner agencies have a strong record of policy development, delivery, and evaluation, plus advocacy at a national and state level, through their own work and via their stakeholder networks. As such, numerous opportunities will be available to advocate for system-level change regarding the way suicide prevention in educational and community settings is approached.

**5.7 How will you share with study participants the progress and findings of your research?**

It is expected that this project will lead to numerous research outputs in the form of a suite of publications in high impact peer-reviewed journals, presentations at national and international conferences, and reports made available via the websites of the Suicide and Self-Harm Research Group, Champs Suicide Prevention, and LJMU. We have costed in for attendance to three significant international and national conferences relevant to the field of young people’s mental health and suicide prevention. Additionally, our research group has a social media page and provides quarterly newsletters where we will continue to report on the outcomes of this study. Bespoke summary reports or leaflets (jointly developed with PPI groups) will be produced for young people, education professionals, policymakers, and parents, and will be shared with all participating schools, to ensure study findings are accessible to all participants.

**6. Project/Research Timetable**

The GANTT Chart (see attached) provides a monthly project timetable showing the scheduling of all key stages in the project. Key milestones include:

Public Advisory Group meetings – three times a year from January 2024

Young Public Advisory Group meetings – three times a year from January 2024

Team management meetings – monthly from January 2024

Trial Steering Committee meetings – six monthly from January 2024

Recruitment of research assistant – 3 months from 1st January 2024.

Start-up phase – 3 months from 1st January 2024.

Recruitment of schools – 6 months from 1st January 2024.

Recruitment of participants - 18 months; March 2024 to September 2025.

Follow-up, process evaluation, final assessments - 20 months; March 2024 - November 2025.

Analysis and report - 3 months; October 2025 to December 2025.

Dissemination activities – 3 months; October 2025 to December 2025.

**7. Project Management**

The PIs, Dr Saini and Dr Ashworth, will oversee and co-ordinate the project start-up, recruitment and data collection and ensure the smooth running of the research. The project manager, Molly McCarthy (1.0FTE, 24 months), will liaise regularly with Co-Investigators to ensure clear lines of communication, manage the development of the website, co-ordinate recruitment of diverse schools, and lead on the writing of study reports. A Research Assistant will be employed (0.6FTE, 18 months) to undertake recruitment activities, research assessments and data collection. They will both work closely with the PIs, and all four will be based at LJMU. A project manager and research assistant should be adequate for meeting the study recruitment target, due to the high level of local support. Research staff will benefit from the research infrastructure developed within LJMU.

Co-applicants forming the TSC includes a statistician, health economist, trial manager, member of the DfE, a Consultant Psychologist from a local CAMHS crisis team, Public Health commissioner, expert academics, researchers, teachers from two schools and one/two members of the PAG and the YPAG, and collaborator Ruth du Plessis who will Chair the group. The TSC will oversee the conduct, progress, and safety of the trial, and will provide advice to the project research team. The TSC will meet at the beginning of the trial and every six months over the four-year study period and will incorporate the safety monitoring committee (SMC). Additional independent members will also be included.

**8. Ethics**

Ethical approval will be obtained from LJMU prior to study commencement. The study will always be undertaken in compliance with the research protocol. All participants will be given a participant information sheet and consent form prior to taking part in interviews. Personal data will be documented in a password protected and encrypted computer. No identifiable patient data will be extracted.

As delivery of the intervention is being arranged by local Public Health bodies, consent will only be sought for completion of the measures. While opt-in gatekeeper consent will be sought from the participating schools, opt-out consent will be sought from parents of YP. Findings from both our scoping and pilot study consistently showed that opt-out consent is feasible and desirable for this project. Given the potentially sensitive nature of the measures, parents/carers will be informed of the project on two separate occasions (via the schools’ usual communication channels), to help ensure information is not missed. Schools will also be asked to advise parents/carers of the date scheduled for survey completion, so they are aware. All parents will be provided with detailed information sheets (alternative format/easy-read will also be developed), outlining the importance of the study, any risk of harm (and procedures put in place to reduce this), and will be provided with detailed signposting. Parents will be able to view the items in the survey if requested and attend an online information session about suicide prevention in young people. Parents and carers who are Co-Is and PPI advisory members will be consulted to ensure 1) improved attendance at the parent information sessions and 2) parents/carers are effectively informed about any young people who may be at risk of suicide and equipped with appropriate resources and support.

Schools will be provided with a detailed support pack for completing the measures with pupils, including age-appropriate lesson plans, PowerPoint slides, and glossary. The slides inform the CYP of the nature of the study and their rights as a participant (including being able to withdraw) and they be delivered to them by the teacher supporting their survey completion. CYP will then be able to indicate if they are happy to proceed by ticking a box at the beginning of the survey. This method has been used successfully in previous trials by EA, in the pilot study, and is recommended in good practice guidance.^38^ For pupils eligible for Reframe IT-UK, they will be provided with an information document/leaflet/video (co-developed with our young person’s advisory group), advising them about the content of Reframe IT-UK, and the voluntary nature of participation. The school’s guidance pack will also remind staff to ensure that pupils are provided information discretely and are made aware that they do not have to take part. Fully informed opt-in consent will be sought for participation in the qualitative strand of the process evaluation. Participants will be verbally reminded of their rights prior to the interviews/focus groups beginning. In case of distress to teachers during MAPSS, the school guidance packs will provide information on promoting staff wellbeing, including details of 24-hour helplines (one specifically for educators, and local NHS crisis lines). In case of distress to parents/carers during MAPSS, the parent/carer participant information sheets will provide details of charities e.g., Papyrus and NHS services including NHS 24-hour crisis helplines.

**9. Patient and Public Involvement** **(PPI)**

The proposed work has been developed as part of the Suicide and Self-Harm Research Group (SSHRG) and with PPI co-applicants including parents and youth worker leads: MJ, TN, DC and LH. Three schools are involved (co-applicant: SY), with teachers and pupils acting as PPI consultants. The ‘3 Dads Walking’ are also collaborators and members of this group. All members have had oversight of the application and have informed its development (e.g., from discussions we have adapted, removed, or replaced some of the measures and adapted components of the MAPSS programme). A young person’s advisory group has been established via Merseyside Youth Association, who are helping us to design information documents/leaflets/videos to empower young people, helping them to understand what both MAPSS and the CBT element will entail, and fully understand their rights throughout the process. MJ, DC, and PS will co-lead and co-ordinate the Public Advisory Group (PAG) for adult members (including parent/carer advisors), while LH, TN and EA the Young People’s Advisory Group (YPAG), to ensure the perspectives of all members are embedded throughout the study. All members will be paid for their time at recommended NIHR PPI rates. To address health inequalities and access to support for young people in suicidal crisis, the Health Inequalities Assessment Toolkit ([www.hiat.org.uk](http://www.hiat.org.uk))^59^ will be used to ensure PPI is embedded throughout the design, measures, and approach used in this study and beyond.

***PPI Leads***

PS and EA, who have 16 years collective experience of conducting PPI in research, will lead the PPI groups alongside PPI co-applicants DC, LH, TN and MJ. PS have worked in research with public advisors for the NIHR ARC Northwest Coast (NWC) for over 10 years and will be able to mentor and train other PPI members including MJ, TN, LH, DC, and other public advisors who join the trial. DC and MJ have both experienced engaging with schools when their children were in a suicidal crisis and have since wanted to influence changes for other parents who may be going through similar difficulties. MJ is from a South Asian community and brings her experience in navigating the system and the barriers to communicating with a school when you are from a different cultural background. DC is a bereaved father of a daughter who died by suicide and the Founder of The Holly Clacy Foundation. DC and the 3 other dads of young women who died by suicide are passionate about suicide prevention being part of the school curriculum and are members of the group. TN and LH work for Merseyside Youth Association (MYA) on delivering mental health lessons for pupils including suicide prevention. Members are located across the country in Chelmsford, Manchester, Liverpool, Cumbria and Norfolk and some have been meeting with the All-Parliamentary Suicide Prevention Group about suicide prevention in schools including a government petition being debated in the Houses of Parliament recently. Funding has been costed for Co-Is via their salary or at the NIHR PPI rates for all the activities they will be involved in. There is also funding included for six young people to be recruited as part of the young people advisory group, which will be supported by MYA.

Further funding of £5000 has been awarded to the PI’s (EA and PS) to develop the advisory groups for both the adults and young people involved in our wider suicide prevention research over the next six months. The funding with pay for their time, PPI leads time to facilitate for each group, and a part-time Coproduction Officer to coordinate and manage this work. Activities will include recruiting additional members, organising and conducting three meetings with each of the groups and two co-design workshops to further develop the implementation of suicide prevention programmes in schools, communities and health care settings. This additional work will further strengthen the PPI activities to be established for this project.

**10. Project/Research Expertise**

**Dr Pooja Saini:** Programme co-lead, supervision of research staff, research group meetings, TSC, co-lead PAGs, editing final reports and leading peer-reviewed papers, liaising with other agencies, developing pathways to impact. Grade 9, 0.15FTE.

**Dr Emma Ashworth:** Programme co-lead, supervision of research staff, research group meetings, TSC, co-lead YPAGs, editing final reports and leading peer-reviewed papers, liaising with other agencies, developing of pathways to impact. Grade 8, 0.15FTE.

**Project Manager (Molly McCarthy):** Project management, TSC, development of project materials and documents, induction/training for PPI, quantitative and qualitative data analysis, write-up of final reports and papers for peer-reviewed journals. Supervised by PS and EA. Grade 7, 1.0FTE.

**Research Assistant:** research meetings, TSC, development of project materials and documents, quantitative and qualitative data analysis, write-up of final reports and peer-reviewed papers. Supervised by the project manager. Grade 6, 1.0FTE.

**Mani Jalota (PPI):** PAG meetings, TSC, provision of an alternative first-hand perspective on parenting children with suicidal ideation and self-harm, data analysis, comments on final reports and peer-reviewed papers and non-academic research outputs. Grade PPI rate, 0.1FTE.

**David Clacy (PPI):** PAG meetings, TSC, provision of a first-hand perspective of a parent bereaved by suicide, data analysis, comments on final reports and peer-reviewed papers and non-academic research outputs. Grade PPI rate, 0.1FTE.

**Damian Hart** (Principal Development Manager): Merseyside Youth Association (MYA) Principal Development Manager, TSC, linking with schools across the region and oversight of MYA staff and YPAG involvement in the trial.

**Prof Neil Humphrey** (Professor of Psychology of Education): expertise in leading large-scale mental health trials in schools, TSC, provide mentoring and input about trial design and conduct in school setting, grant management more widely. Grade 10, 0.02FTE.

**Prof. Jo Robinson** (MAPSS Australia lead): TSC, provide mentoring and input about trial design and conduct, grant management more widely, knowledge of MAPSS Australia and development of Reframe, provision of Reframe IT licence. Grade 10, 0.02FTE.

**Dr Samuel McKay** (MAPSS Australia project manager): TSC, will provide mentoring and input about trial design and conduct, and project management of MAPSS Australia. Provides knowledge of MAPSS Australia and development of Reframe. Grade 7, 0.05FTE.

**Sian York** (Deputy Headteacher and Safeguarding Lead): TSC, linking in with schools, comments on final reports and peer-reviewed papers, liaising with other agencies, development of pathways to impact. Grade Deputy Head Teacher, 0.05FTE.

**Dr Vivienne Crosbie** (Clinical Lead of Alder Hey Hospital’s CAMHS crisis team): TSC, supporting liaison with schools, considering clinical implications of work, and translation of project findings into guidelines and policy. Grade 10, 0.05FTE.

**Dr Steven Lane** (statistician): TSC and research meetings, provide expertise in evaluating quantitative data. Grade 9, 0.05FTE.

**Prof Gerry Richardson** (health economist): TSC and research meetings, provide expertise in evaluating the health economics of SH interventions. Grade 10, 0.05FTE.

**Neil Boardman** (Cheshire and Merseyside Public Health Collaborative): commissioning of interventions, TSC, provide public health support for this project within his role as Suicide Prevention Programme Manager, support school recruitment and support with the commissioning of the MAPSS program in a future trial. Grade Public Health Lead, 0.05FTE.

**Kate Henderson** (CYP Cognitive Behavioural Therapist): TSC, linking in with schools, comments on final reports and peer-reviewed papers, liaising with other agencies, development of pathways to impact. Grade Cognitive Behavioural Therapist, 0.05FTE.

**Dr Maria Michail:** research meetings, TSC, development of project materials and documents, quantitative data analysis, write-up of final reports and peer-reviewed papers. Supervised by the project manager. Grade 9, 0.05FTE

**Christopher Price** (collaborator): TSC, linking with schools across the region as part of his current role as Local Authority school relationship manager.

**Mike Palmer, Andy Airey, Tim Owen** (collaborators): Three dads walking. PAG meetings, support by providing an alternative, first-hand perspective on parenting child with suicidal ideation and bereaved by suicide.

**Celia Mae Jones** (Collaborator): Grassroots Suicide Prevention Charity Manager. TSC. Management of the delivery of suicide prevention lessons in schools.

**James Parkes** (Collaborator): Papyrus regional manager North West. TSC. Management of the delivery of SP-OT and SP-ARK online training.

**Lori Hawthorn** (Collaborator): Public Health Improvement Officer. Developing safety plans with CYP for schools. Support YPAG development and management as has links with schools across Cheshire. External Committee member for TSC.

**Ruth du Plessis** (Collaborator): Director of Public Health and lead commissioner for funding MAPPS SP-OT and SP-ARK training and suicide awareness lessons. External Chair for TSC.

**Catherine Newsome** (Collaborator): Head of Health and Wellbeing Analysis and Research Department of Education, External Committee member of the TSC and will disseminate findings within educational policies.

**Applied Research Collaborative North West Coast (ARC NWC):** Working with the team as partners in the project and will provide support with PPI training.

**Deryn Hanmer** (collaborator): Martin Gallier Project, a third sector organisation suicide prevention provider will be supporting schools with suicide prevention lessons and providing community support. External Committee member for TSC.

**Leigh Horner** (Merseyside Youth Association Project Co-Ordinator): YPAG meetings, research meetings, supporting young people in YPAG, data analysis, comments on final reports, peer-reviewed papers and non-academic research outputs, timescale 0.1FTE

**Tony Niemen** (Merseyside Youth Association Mental Health Promotion Worker): YPAG meetings, research meetings, supporting young people in YPAG, data analysis, comments on final reports, peer-reviewed papers and non-academic research outputs, timescale 0.1FTE

Collaborators from the regional **Integrated Care System (ICS)** are also involved.

**11. Success Criteria and Barriers to Proposed Work**

Success of the trial will be judged based on the following indicators:

- Meeting planned targets for recruitment of schools and pupils into the trial
- Retention of participants in the trial
- Collection of qualitative and quantitative outcome data from eligible participants, including drop-out participants.

The proposed trial is part of a wider programme of research with the aim of establishing the efficacy of MAPSS and supporting its translation into schools nationwide. Funding for a future trial would be sought from NIHR Public Health Research. The transition to the efficacy RCT will be supported by a series of workshops involving teachers, health professionals, researchers and those personally affected by suicide to review the findings of the feasibility RCT and support the design of the definitive study. Workshops will explore how MAPSS could be effectively implemented into schools, should it prove to be efficacious, and identify barriers to implementation. NPT^57^ will be used as a framework to guide the translation of MAPSS into routine use in schools with the support of trained staff. A PAG and YPAG for MAPSS includes individuals with experience of suicidal behaviours either themselves or as a carer/parent. This group will run beyond the life of this project to support the transition to the larger efficacy RCT. Group members will receive training in research methods and practice via our project partners the NIHR ARC NWC to support capacity building within the advisory groups.

A key risk to meeting recruitment targets will be if a lack of either school or pupil motivation means that too few participants are recruited. Our consultation with potential recruitment sites (schools), members of the Local Authority, teachers, pupils, and health professionals indicate that study sites, teachers, and eligible pupils would be motivated to engage with this study. Moreover, a large number of potential additional recruitment sites exist within the region alone. Recruitment rates will be regularly monitored against expected monthly targets. If it appears that recruitment rates are lower than planned, we will expand the number of recruitment sites.

Participant retention may be affected due to inconvenience or inaccessibility of the online intervention, or lack of perceived benefit. The school-based setting of MAPSS, regular telephone contact and reminders, and meetings scheduled in participants’ schools will all help support retention. A regular study newsletter will also help emphasise the importance of the trial and help participants feel a valued part of the research. Where participants do drop out, efforts will be made to undertake an exit interview to ascertain the main reasons for this. This information can then be used to mitigate against further drop out (e.g., adjusting level of researcher contact where a lack of contact leads to disengagement).

A further key risk to collecting outcome data will be if participants feel overburdened by the number of assessments. The choice and number of assessments have been streamlined as far as possible and reviewed as part of project PPI activities and scoping study, and have been deemed acceptable. Where participants feel over-burdened, steps such as breaks in interviews will be adopted to make the load more manageable. Surveys for staff will be designed to be as short as possible, and can be completed at a time to suit them.
